# Mapping of Adenovirus of serotype 3 fibre interaction to desmoglein 2 revealed a novel ‘non-classical’ mechanism of viral receptor engagement

**DOI:** 10.1038/s41598-018-26871-x

**Published:** 2018-05-30

**Authors:** Emilie Vassal-Stermann, Manon Mottet, Corinne Ducournau, Frédéric Iseni, Charles Vragniau, Hongjie Wang, Chloe Zubieta, André Lieber, Pascal Fender

**Affiliations:** 1 Institut de Biologie Structurale (IBS), Université Grenoble Alpes, CNRS, CEA, 71 Avenue des Martyrs, 38042 Grenoble, France; 2 Unité de Virologie, Institut de Recherche Biomédicale des Armées, BP 73, 91223, Brétigny-sur-Orge Cedex, France; 3 University of Washington, Department of Medicine, Division of Medical Genetics, Box 357720, Seattle, WA 98195 USA; 4 Laboratoire de Physiologie Cellulaire et Végétale, Biosciences and Biotechnology Institute of Grenoble, UMR5168, CNRS/CEA/INRA/UGA, 17 Rue des Martyrs, 38054 Grenoble, France

## Abstract

High-affinity binding of the trimeric fibre protein to a cell surface primary receptor is a common feature shared by all adenovirus serotypes. Recently, a long elusive species B adenovirus receptor has been identified. Desmoglein 2 (DSG2) a component of desmosomal junction, has been reported to interact at high affinity with Human adenoviruses HAd3, HAd7, HAd11 and HAd14. Little is known with respect to the molecular interactions of adenovirus fibre with the DSG2 ectodomain. By using different DSG2 ectodomain constructs and biochemical and biophysical experiments, we report that the third extracellular cadherin domain (EC3) of DSG2 is critical for HAd3 fibre binding. Unexpectedly, stoichiometry studies using multi-angle laser light scattering (MALLS) and analytical ultra-centrifugation (AUC) revealed a non-classical 1:1 interaction (one DSG2 per trimeric fibre), thus differentiating ‘DSG2-interacting’ adenoviruses from other protein receptor interacting adenoviruses in their infection strategy.

## Introduction

Adenoviruses (Ads) are a family of non-enveloped double-stranded DNA viruses infecting a wide variety of hosts including mammals. Infection occurs through a sequential two-step cell internalization process involving the binding of the adenovirus fibre protein to an attachment receptor followed by the interaction of the penton base protein with integrins that trigger endocytosis^1^. Different species of adenoviruses, including human adenoviruses, use distinct receptors for infection.

Human adenovirus serotypes (HAds) have been classified into seven species (A to G). The fibre of multiple serotypes from species A, C, D, E and F (*e*.*g*. serotypes 2, 4, 5, 9, 12, 15, 19, 31, 41) has been described to use the coxsackie and adenovirus receptor (CAR) as a primary attachment receptor^2,3^. However, species B adenoviruses use either CD46 or desmoglein-2 (DSG2) as a receptor^4–6^. For instance, HAd16, HAd21, HAd35 and HAd50 nearly exclusively use CD46 as a receptor whereas HAd3, HAd7, HAd11 and HAd14 share the newly identified DSG2 receptor. Moreover, HAd11 and to a lesser extend HAd3 interact with both CD46 and DSG2 based on receptor density at the cell surface^6,7^. The identification of this new DSG2 receptor was made using a HAd3 virus-like-particle (VLP) named Penton Dodecahedron (Pt-Dd). This particle is composed of twelve penton bases interacting together in a symmetric manner with twelve fibres protruding from the particle surface. Pt-Dd is produced during the natural Ad3 replication cycle but can also be recombinantly expressed using the baculovirus system^8,9^. In agreement with previous observations made for CAR and CD46, the interaction of Pt-Dt with DSG2 involves specifically the distal globular part of the adenovirus fibre called the knob domain^10–13^.

DSG2 is mainly found in epithelial cells but also in cardiomyocytes where mutations are responsible for arrhythmogenic right ventricular dysplasia and cardiomyopathy^14^. In desmosomes, DSG2 receptors are in a strong heterophilic interaction with Desmocollin 2 (DSC2)^15^. Both DSC2 and DSG2 belong to the type-1 transmembrane proteins of the cadherin superfamily and interact together through their distal extracellular cadherin domain, called EC1. The whole ectodomain of DSG2 is composed of four extracellular cadherin domains, EC1 to EC4, and a domain proximal to the membrane, the Extracellular Anchor (EA). The crystallographic structure of DSG2 has recently been published^16^. Each EC domain carries N-glycosylations with EC4 having an extra O-glycosylation. The linearity of DSG2 ectodomain is drastically disrupted by a 100° hinge between EC3 and EC4.

Interestingly, DSG2 expression can vary in different cancer types. For instance, overexpression of this receptor has been reported in non-small cell lung cancer or in squamous cell carcinoma correlating with a high risk of metastasis^17,18^. Indeed, cancer cells maintain strong intercellular junctions such as tight junctions, adherens junctions, gap junctions and desmosomes, which limit the diffusion of drugs in tumours^19,20^. It has been described that the simultaneous interaction of several knob domains with DSG2, a situation found in the whole virus or in Pt-Dd, which presents 12 fibres per particle, triggers intracellular signalling, and in turn cell membrane remodelling^9,21,22^. This property has been exploited to develop “junction openers” (JOs) which enhance the efficacy of cancer drug compounds such as MAbs or DOXIL^®^^23,24^. The binding of JOs to DSG2 receptor is responsible for transient epithelial-to-mesenchymal transition (EMT) and DSG2 shedding^6,25,26^, which partially unlocks these junction padlocks.

Since Adenovirus is the most used vector in human gene therapy trials, deciphering the molecular basis of ‘DSG2-interacting’ HAd-fibres binding to DSG2 may create a new basis for the development of next-generation therapeutics. Indeed, rational design of mutated fibre for detargeting/retargeting could be developed for DSG2-interacting oncolytic adenovectors^27–29^. Moreover, beside this vector-based approach, a better comprehension of the HAd/DSG2 interaction would enable the design of improved JO molecules, thus potentiating the therapeutic efficacy of approved chemotherapeutic drugs or therapeutic MAbs. In the present work, we have mapped the critical EC domain required for HAd3 binding to DSG2. In addition, based on the stoichiometry of the measured interactions, a non-classical mechanism of HAd3 fibre binding to DSG2 is reported. This interaction is a novel binding strategy not observed in other adenovirus/protein receptor complexes such as Ad/CAR and Ad/CD46.

## Results

### Production of glycosylated EC1-4 desmoglein 2

**Figure 1:**
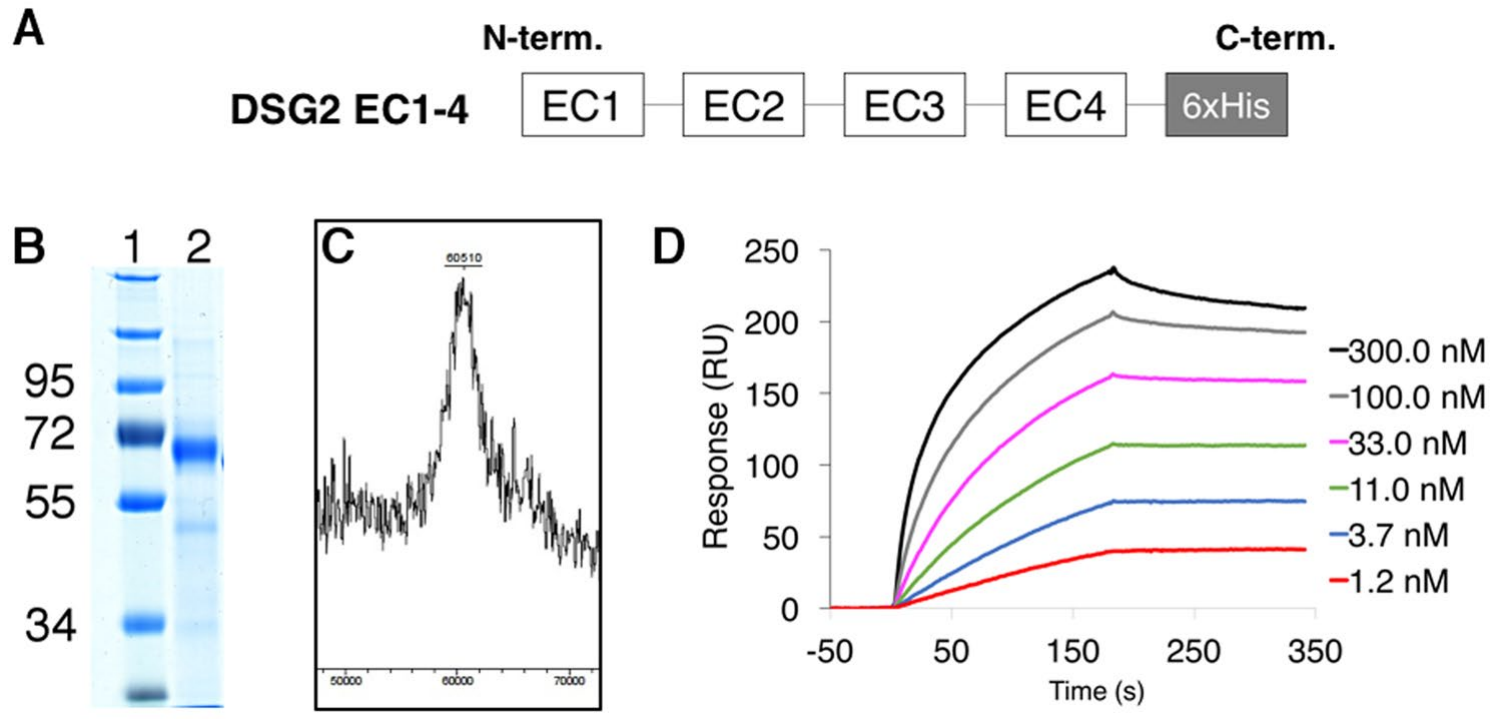
General characterization of the DSG2 full length ectodomain. (**A**) Schematic representation of recombinant DSG2 containing the four extracellular subdomains (EC1-4) and a C-terminal His tag. (**B**) InstantBlue-stained SDS-PAGE of purified recombinant DSG2 EC1-4; full-length gel is presented in Supplementary Figure S1. (**C**) MALDI-TOF mass spectrum of DSG2 EC1-4. (**D**) SPR analysis of the interaction of PtDd with immobilized recombinant DSG2 EC1-4. PtDd was injected at different concentrations ranging from 1.2 to 300 nM.

To ensure that DSG2 ectodomain can be produced as a functional protein, we first expressed the whole ectodomain carrying the four cadherin domains (EC1-4) with a C-terminal His-Tag (Fig. 1A). To as closely as possible mimic what is found at the cell surface, we used the mammalian HEK-293 F expression system to produce secreted EC1-4. This resulted in the production of a highly glycosylated native protein. After purification, the protein migrated on SDS-PAGE gel about 10 KDa above its predicted mass (51 KDa), reflecting the presence of glycosylation (Fig. 1B). Furthermore, the molecular mass was determined more accurately by MALDI TOF and a peak around 60.5 kDa confirmed the presence of more than 9 kDa of glycans, in accordance with recently published results^16^ (Fig. 1C). Since we have previously shown that HAd3 recombinant penton-dodecahedron (Pt-Dd), a dodecahedric particle with twelve fibres, interacts with a commercial DSG2 by surface plasmon resonance (SPR) experiments^6^, the functionality of recombinant DSG2 EC1-4 was checked using the same experimental set-up. In contrast to the fibreless ‘base-dodecahedron’ particles (Bs-Dd) (not shown), Pt-Dd interacted with DSG-2 in a dose-dependent manner at nanomolar concentrations (Fig. 1D).

### Mapping of DSG2 binding domains

#### Co-elution chromatography studies

**Figure 2:**
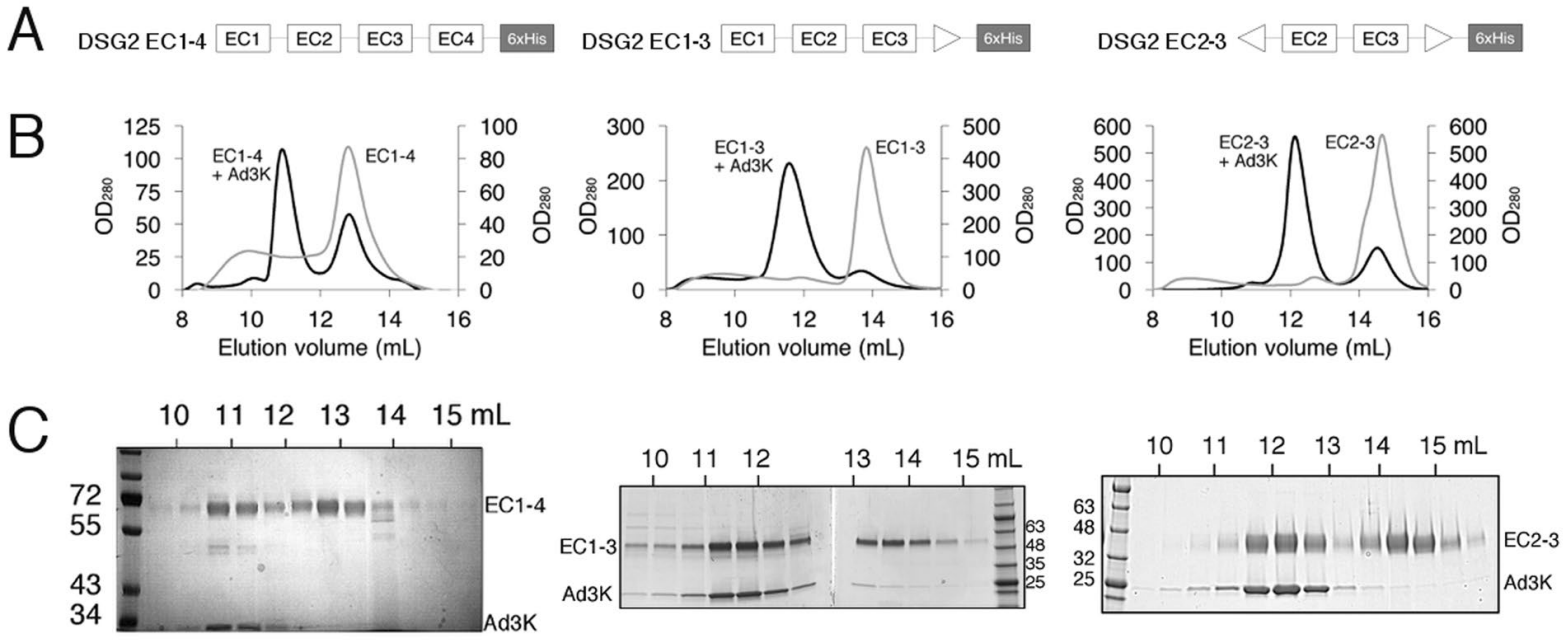
Mapping of EC domains required for fibre binding (**A**) Schematic representation of the different recombinant DSG2 constructs EC1-4, EC1-3 and EC2-3. (**B**) Gel filtration based co-elution experiments. Size exclusion chromatography analysis of Ad3K co-incubated with the different constructs demonstrates a shift of elution to smaller retention volumes in each case thus reflecting an increased molecular weight. This is an agreement with a complex formation between DSG2 and Ad3K. (**C**) 10 or 4–20% SDS-PAGE analysis of the peak fractions aligned to its corresponding chromatogram show the presence of both Ad3K and DSG2 construct (as indicated) in the shifted peak confirming the formation of a complex.

We next attempted to identify DSG2 cadherin domains that are critical for recombinant Ad3 fibre knob (Ad3K) binding by using co-elution chromatography studies. We compared the chromatographic profiles of purified recombinant DSG2 domains (Fig. 2A) in the presence or absence of Ad3K by size exclusion chromatography. Unbound DSG2 EC1-4 eluted as a single peak with an elution volume of 12.8 mL whereas DSG2 EC1-4 in complex with Ad3K eluted earlier at 10.9 ml, suggesting complex formation (Fig. 2B, left panel). SDS-PAGE analysis of the peak 1 (10 to 12 mL) confirmed co-elution of Ad3K and DSG2 EC1-4 (band below 34 and above 55 kDa respectively in Fig. 2C). Fractions corresponding to peak 2 (12 to 14 mL) clearly showed an excess of DSG2 EC1-4.

To test the involvement of the EC4 domain in DSG2 binding, we generated an EC4-deleted DSG2, named DSG2 EC1-3 by transient transfection in HEK-293 F (Fig. 2A, middle panel). Co-elution chromatography experiments showed that DSG2 EC1-3 bound efficiently to Ad3K (Fig. 2B, middle panel). This suggests that the EC4 domain does not contribute to binding with Ad3K. We also produced DSG2 in which the EC1 and the EC4 domains were both deleted (EC2-3) (Fig. 2A, right panel). A similar elution profile was observed, with a first peak corresponding to the complex EC2-3/Ad3K (11 to 13 mL) and a second peak corresponding to an excess of EC2-3 (13 to 15 mL). These results indicate that, although the EC1 domain is needed for the strand-swap mechanism of adhesive binding in classical cadherins, it is not essential for optimal Ad3K binding. The EC2-3 cadherin ectodomain fragment is thus sufficient for the interaction with Ad3K.

#### Minimal DSG2 domain determination by Surface Plasmon Resonance (SPR)

**Figure 3:**
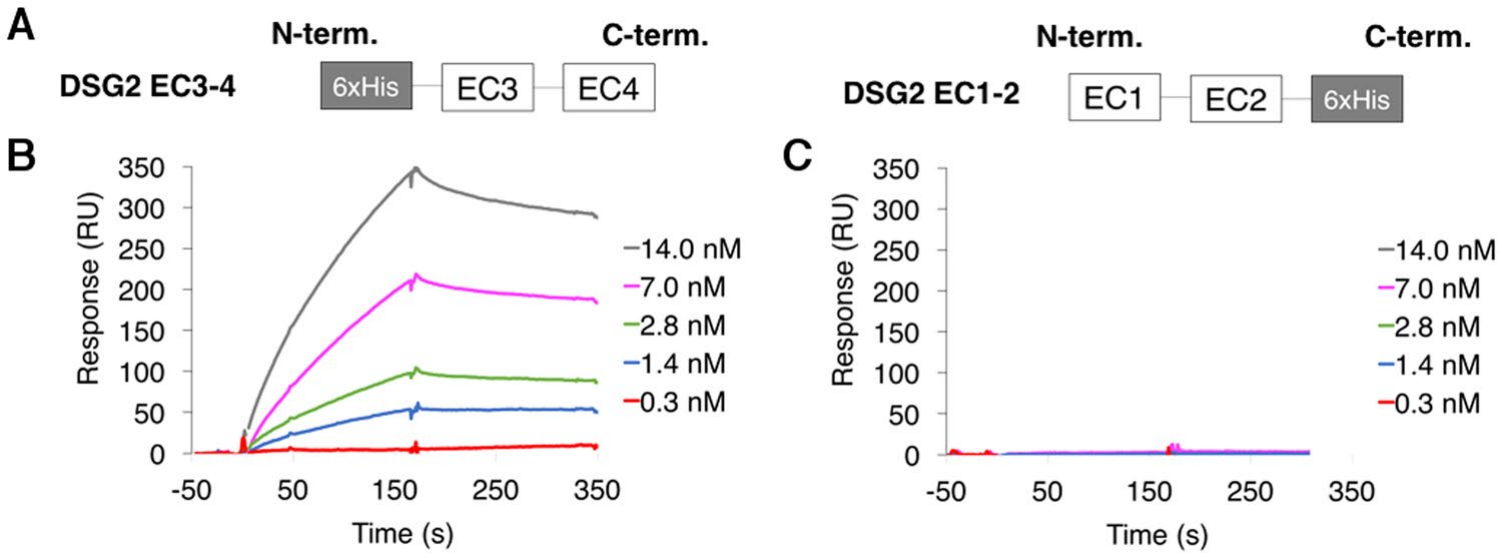
Determination of the interactions by EC2 or EC3 by SPR. (**A**) Schematic representation of recombinant DSG2 constructs EC1-2 and EC3-4. (**B**) SPR analysis of the interaction of PtDd with immobilized DSG2 EC3-4. Pt-Dd was injected at different concentrations ranging from 0.3 nM to 14.0 nM. (**C**) Same analysis as in (**B**) with DSG2 EC1-2.

The goal of this experiment was to more precisely distinguish the respective roles of EC2 and EC3 in the interaction with Ad3K. Despite extensive efforts to produce EC3 or EC2 constructs in the HEK-293 system, we did not manage to express these domains alone as soluble proteins. We then decided to split the DSG2 ectodomain in EC1-EC2 and EC3-EC4 (Fig. 3A). Of note, the recombinant DSG2-cadherin EC3-4 fragment was produced in bacteria and thus lacks glycosylation.

The concentration-dependent binding of Pt-Dd to immobilized DSG2 EC3-4 (Fig. 3B) indicates that EC3 is sufficient to mediate the interaction. Conversely, Pt-Dd showed no interaction at all to immobilized DSG2 EC1-2 (Fig. 3C). EC3 domain is thus critical for Ad3K binding and since SPR is a very sensitive method to detect even weak interactions, this also means that EC2 alone is not able to mediate this interaction. Moreover, the surface was regenerated by injection of 10 mM EDTA, indicating that the EC3 cadherin domains must be loaded with calcium to enable this specific interaction.

Altogether, these data demonstrate that EC3 domain is specifically required for fibre binding and that the lack of glycosylation in these constructs produced in *E*. *coli* did not impair binding thus supporting the de-glycosylation experiments shown in Supplementary Fig.S1. Since these post-translational modifications of DSG2 are not required for the interaction it can be concluded that Ad3 fibre knobs interacts with the EC3 polypeptide in a calcium dependent manner and not with glycans.

### Co-expression experiments

**Figure 4:**
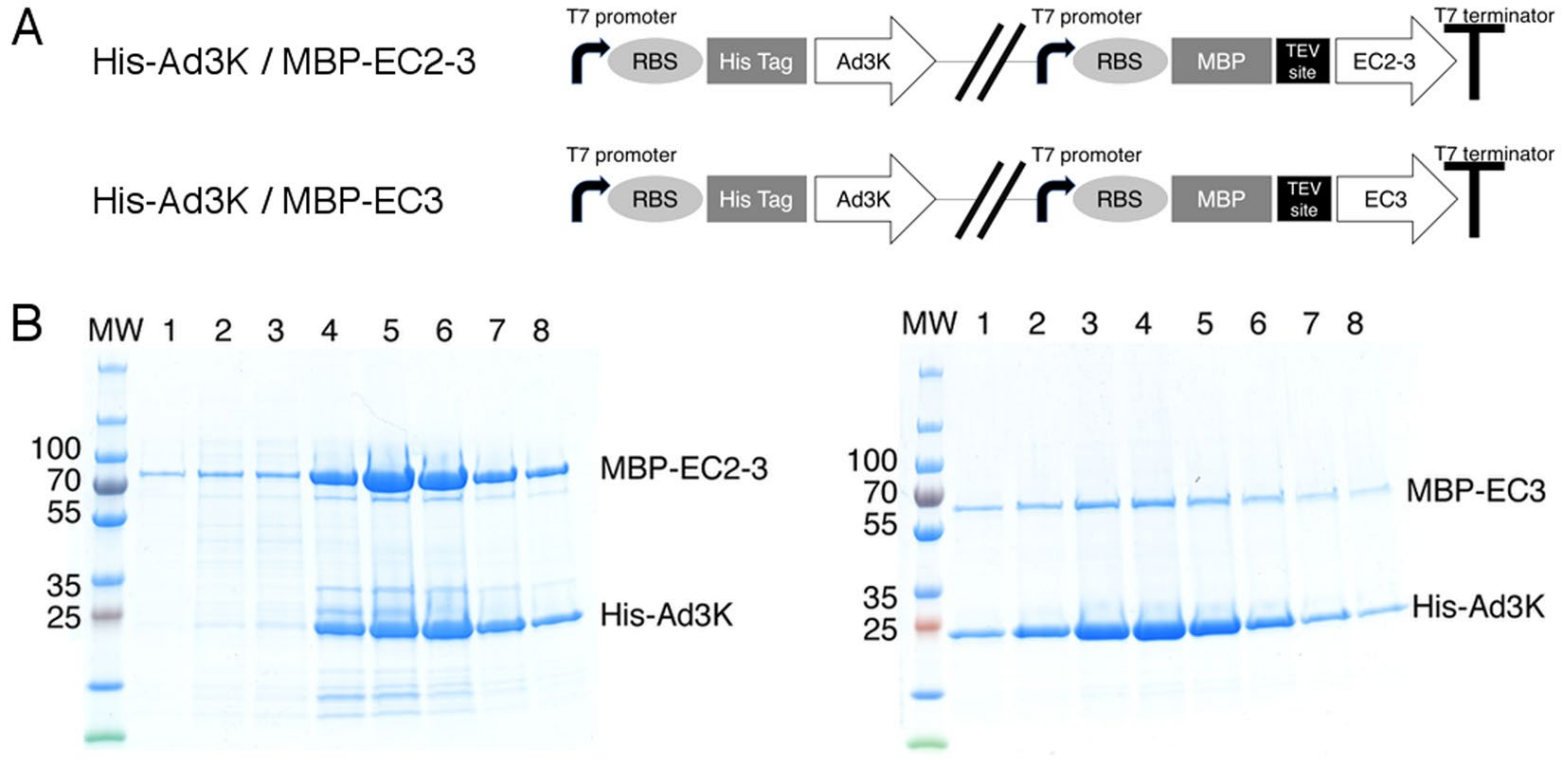
Prokaryotic coexpression of the complex. (**A**) Schematic representation of DSG2/Ad3K pETDuet vectors. RBS: Ribosome Binding Site; MBP: Maltose Binding Protein tag. (**B**) Affinity purification of recombinant HisAd3K and associated proteins from *E*. *coli* extracts. pETDuet vectors expressing His-Ad3K and MBP-EC2-3 or MBP-EC3 were grown and induced with IPTG. Cells were lysed and insoluble material was removed by centrifugation. His Ad3K was purified by nickel affinity chromatography as described in Experimental Procedures. Sample contents from elution fractions 1–8 were analyzed by SDS-PAGE. Left panel shows the MBP-EC2-3 coelution with His-Ad3K, right panel shows the same experiment with MBP-EC3.

In order to provide additional support for these results and to further characterize the complex, we co-expressed the N-terminal His-tagged Ad3K (His-Ad3K) together with DSG2 EC2-3 or DSG2 EC3 in bacteria. Given that expression of DSG2 in bacteria showed relatively low yields of soluble protein, we fused the DSG2 domains to the Maltose Binding Protein (MBP) to increase solubility with an upstream Tobacco Etch Virus (TEV) protease cleavage site (Fig. 4A) to allow the subsequent removal of the MBP tag prior to biophysical studies.

Lysates of either His-Ad3K and MBP-DSG2 EC2-3 or His-Ad3K and MBP-DSG2 EC3 were loaded onto a His GraviTrap column and eluted with imidazole. SDS-PAGE analysis showed that both MBP-DSG2 EC2-3 (theoretical mass 69.9 kDa) and MBP-DSG2 EC3 (theoretical mass 57.3 kDa) co-eluted with His-Ad3K (theoretical mass 23 kDa), demonstrating complex formation between the two proteins (Fig. 4B, left and right panels, respectively). This result indicates that EC3 domain alone is sufficient to interact with Ad3K and that EC3 is the critical domain of DSG2 targeted by the viral protein.

### TEM imaging of the complex after MBP removal

**Figure 5:**
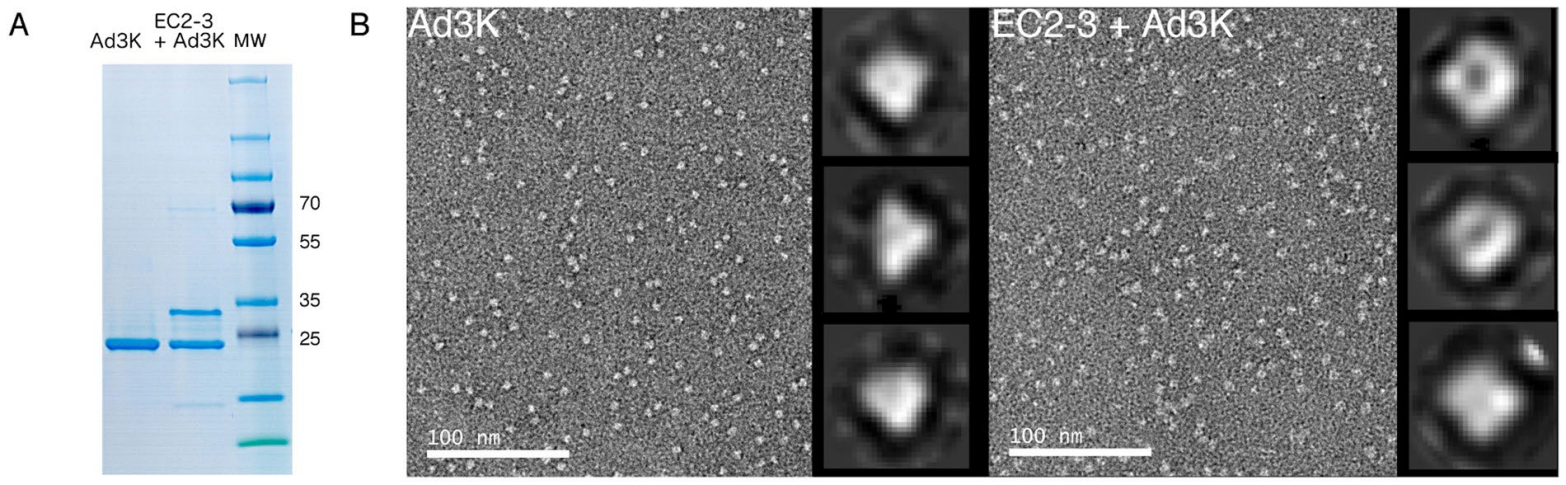
Purification and electron microscopy of MBP-free Ad3K + EC2-3 complex. (**A**) InstantBlue-stained SDS-PAGE of the purified Ad3K and Ad3K + EC2-3 complex after MBP removal. Full-length gel is presented in Supplementary Figure S2. (**B**) Negative-staining electron microscopy analysis. Left panel: Electron micrograph of Ad3K stained with uranyl acetate. Right panel: Electron micrograph of Ad3K in complex with EC2-3 stained with uranyl acetate. Representative class averages are shown on the galleries to the right of each micrograph.

The stability of these two complexes after MBP removal was then investigated. After TEV protease treatment, complexes were loaded again on the His GraviTrap column and then purified by Size Exclusion Chromatography (SEC). Digested DSG2 EC2-3 (theoretical mass 27.8 kDa) and His-Ad3K still co-eluted during this second purification steps (Fig. 5A), reflecting the stability of this MBP-free complex. Conversely, no co-elution was observed for Ad3K in complex with EC3 domain alone after MBP removal (not shown). Altogether, this data indicates that (*i*) EC3 is the domain targeted by Ad3K for binding (*ii*) complex formation is successful by co-expression of His-Ad3K and receptor constructs in bacteria and (*iii*) EC3 required a stabilizing peptide such as MBP or the EC2 domain to be functional. Based on these data, EC2-3 was used for the next experiments aiming at investigating the oligomeric status of the complex.

The homogeneity of Ad3K-EC2-3 complex was then studied by negative stain electron microscopy and compared to Ad3K alone. While both samples appeared homogenous, the particles corresponding to the complex were distinct from Ad3K alone (Fig. 5B). The shape was altered by the presence of the receptor on the trimeric knob. Since these objects are very small, they were subjected to class averages (representative classes are enlarged on the right of the corresponding micrographs). As expected, Ad3K alone gave triangular shapes whereas the three-fold symmetry was lost when receptor was added. Neither triangular nor hexagonal shapes were found for the latter, suggesting that the stoichiometry might be different from that reported for other adenovirus serotypes interacting with proteinaceous receptors (*i*.*e*. three receptors per trimeric knob)^11,12^. To confirm this, additional biophysical experiments were performed.

### Stoichiometry determination

#### Multi-Angle Laser Light Scattering (MALLS experiments)

**Figure 6:**
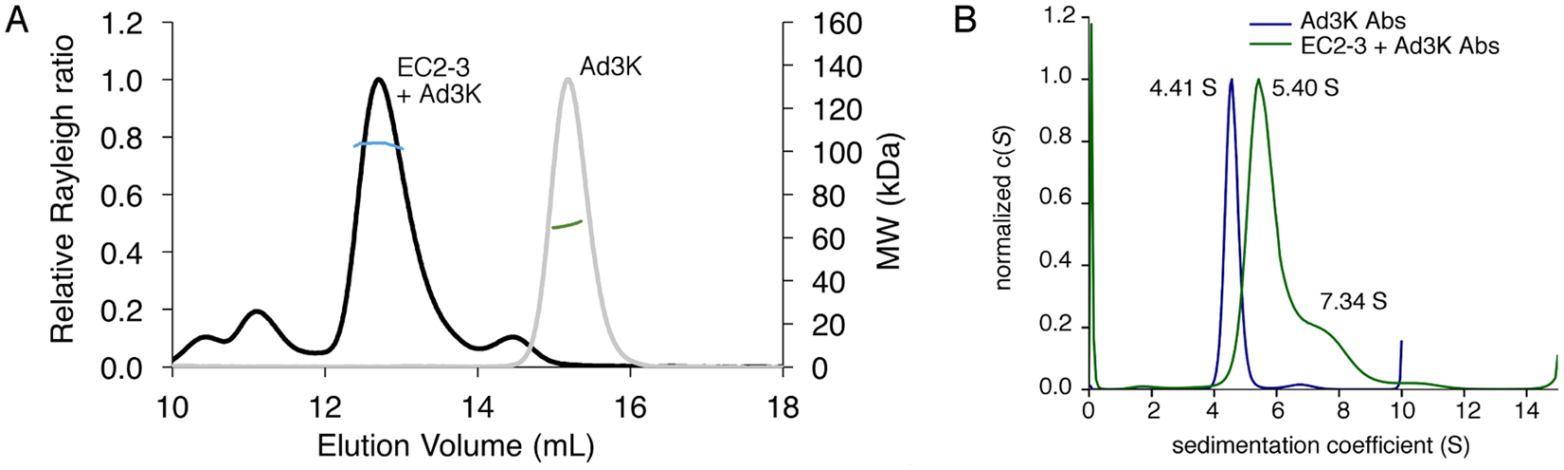
Stoichiometry analysis of the Ad3K + EC2-3 complex. (**A**) Molecular mass determination by SEC-MALLS. Overlay of SEC elution profiles of Ad3K (grey line) and Ad3K in complex with EC2-3 (black line). The lines in green or blue represent the average molecular weight of the major Ad3K and Ad3K + EC2-3 species calculated by using the light-scattering measurements (scale on the right y axis). (**B**) Analytical ultracentrifugation (AUC) analysis. Normalized c(s) distribution curves calculated from the raw data using SEDFIT are shown for Ad3K (in blue) and for Ad3K in complex with EC2-3 (in green). The resulting sedimentation coefficients are indicated for the main peaks.

To investigate the oligomeric state of the His-Ad3K/DSG2 EC2-3 complex in solution, SEC coupled to Multi-Angle Laser Light Scattering (SEC-MALLS) was performed to estimate its molecular weight as compared to His-Ad3K alone (Fig. 6A). The data indicated a molecular weight of ∼66 kDa with a 1.3% fitting error and a polydispersity of 1.000 for the trimeric His-Ad3K (theoretical mass is 69 kDa) and a molecular weight of ∼103 kDa with a 1.8% fitting error and a polydispersity of 1.000 for the His-Ad3K/DSG2 EC2-3 complex. This latter value is consistent with a single monomer of DSG2 EC2-3 binding a trimer of His-Ad3K (expected mass 96 kDa) and is too low to reflect two or three receptor molecules binding a trimer of His-Ad3K (expected mass 120 kDa and 144 kDa, respectively).

#### Sedimentation Equilibrium- Analytical Ultra Centrifugation (SE-AUC experiments)

Sedimentation equilibrium analytical ultra-centrifugation (SE-AUC) was then used to further elucidate the stoichiometry of the complex in solution (Fig. 6B). Data obtained by SE-AUC for His-Ad3K were in good agreement with those from SEC-MALLS, showing a sedimentation coefficient of 4.41 S corresponding to a mass of approximately 69.6 kDa, which are consistent with the predicted masses of the trimeric knob.

With respect to the His-Ad3K/DSG2 EC2-3 complex, AUC revealed one major peak representing 72% of the signal, with sedimentation coefficients of 5.40 S corresponding to a mass of approximately 95.1 kDa. This value is in agreement with the one receptor per trimeric knob scenario (expected mass 96 kDa). This peak was followed by a shoulder representing 19% of the signal, with sedimentation coefficients of 7.34 S, corresponding to a mass of approximately 132.4 kDa. While it is difficult to attribute this minor peak, its sedimentation coefficient rules out a three receptor per trimeric knob scenario. By combining the SEC-MALLS and SE-AUC data, it can be concluded that the main species in solution corresponds to a homogeneous complex made of one trimeric knob bound to a single EC2-3 receptor.

## Discussion

Intuitively, one can imagine that viruses evolve to interact with well-displayed receptors at the apical side of epithelial cells. Paradoxically, a number of viruses have been reported to interact with poorly accessible receptors hidden at the cell-cell junction. Among them, both enveloped and non-enveloped viruses have been identified such as Measles Virus, Herpes Simplex Virus, Hepatitis C Virus, Reovirus and Cocksackievirus (for review, see Mateo *et al*.^30^). Adenoviruses are another example of this since the first identified and most common adenovirus receptor, CAR, is a member of tight junctions^2,31^. In our work, we have investigated the molecular interaction of the Human species B adenovirus, HAd3, to the junctional component, DSG2. This latter is naturally found in heterophilic interaction with desmocollin2 (DSC2) to form desmosomal junctions between epithelial cells^32^. DSG2 expands the repertoire of cell-cell junction receptors that can be targeted by adenovirus.

The structure of DSG2 has been recently solved by X-ray crystallography revealing that N-glycosylations are linked to each EC domain, thus confirming bio-informatics predictions^16^. Based on our current study, we have expressed DSG2 ectodomain in mammalian cells in order to preserve the glycosylation pattern of the protein. DSG2 constructs were cloned in the pHLsec plasmid enabling their maturation in the endoplasmic reticulum and Golgi apparatus before being secreted in the medium. As expected, the whole ectodomain construct (EC1-4), with a theoretical MW of 51 kDa, was glycosylated and ran as a relatively diffuse band in SDS-PAGE (Fig. 1B). Mass spectroscopy analysis confirmed the numerous post-translational modifications of this construct as reflected by the wide peak displaying an average mass of 60.5 kDa (Fig. 1C). This highly-glycosylated DSG2 ectodomain was functional as shown by the SPR experiments in which increasing amounts of Pt-Dd were injected over the immobilized receptor (Fig. 1D). Of note, sensorchip regeneration was performed with EDTA, reflecting that calcium was required for the proper folding of EC1-4.

The atomic structure of the DSG2 ectodomain has also revealed that the protein can make homophilic interactions via its distal EC1 domain at least under crystallisation conditions^16^. However, this putative dimerization of DSG2 is not a requirement for Ad binding, since EC1-containing (EC1-4, EC1-3) as well as EC1-devoid constructs (EC2-3) were equally able to form a stable complex with Ad3K as demonstrated by our SEC experiments (Fig. 2B). Since EC2-3 carried two cadherin domains shared by all three constructs, we tried to define more precisely which of these extracellular domains was critical for the interaction with Ad3K. Thus, EC1-2 and EC3-4 constructs were produced and characterized by SPR for Ad3K binding. No binding was detected for EC1-2 by this sensitive method indicating that domain 2 was not sufficient to enable adenovirus binding and suggested that EC3 is the critical domain required for this interaction. This observation was confirmed by the SPR experiments using the EC3-4 construct, which showed a dose-dependent binding of Ad3K (Fig. 3B). Taken together, EC3 is the main domain of DSG2 targeted by HAd3. This result is in agreement with an experiment described by Wang et *al*., which reported that 6D8 and 8E5 MAbs (directed against EC3 and EC3-4 respectively) were able to decrease by 20 to 40% HAd3 binding to the cell surface (and up to 65% when used in combination) while other MAbs directed against EC1 or EC2 did not compete at all^6^.

Since the EC3-4 construct produced in a prokaryotic system was functional (Fig. 3B), this allowed prokaryotic coexpression of Ad3K with different DSG2 constructs. In a first attempt, EC2-3 was cloned in fusion to a MBP tag and co-expressed in pETDuet vector with 6X His-tagged Ad3K. Immobilized Metal-Affinity Chromatography (IMAC) purification clearly showed that MBP-EC2-3 was retained by the His-tagged Ad3K (Fig. 4B) thus demonstrating that glycosylation is not required for the Ad3K-DSG2 interaction as further demonstrated by the de-glycosylation experiments shown in Supplementary Fig. S1. A similar experiment was performed with the EC3 domain alone fused to MBP and gave the same result, confirming that EC3 is sufficient to make a stable complex with Ad3K. To further characterise the complex, MBP was removed *via* a TEV cleavage site. Removal of MBP did not affect EC2-3/Ad3K complex formation as seen by both SDS-PAGE (Fig. 5A) and electron microscopy (Fig. 5B). However, complexes with only EC3 were unstable upon MBP cleavage (not shown). This result indicates that even if EC3 is sufficient for Ad3K binding, it likely needs to be fused to a solubilizing polypeptide such as EC2 or MBP, at least in prokaryotic expression systems. In the absence of these polypeptides, the potential ‘chaperone-like’ activity played by Ad3K co-expressed with EC3 alone is not sufficient to stabilize the complex.

Since MBP tends to multimerize, it is necessary to remove this tag to unambiguously analyse the stoichiometry of the complex. To study the stoichiometry, Ad3K/MBP-EC2-3 complex was purified by IMAC, cleaved by TEV protease and MBP-free Ad3K/EC2-3 complex was recovered using a second IMAC as shown in Fig. 5A. This latter complex was then analysed by MALLS (Multi-Angle Laser Light Scattering), which enables the determination of the molecular weight of molecules or complex separated by SEC. Molecular weight and dispersity were assessed from the main peak (Fig. 6A) indicating that the complex was monodisperse (1.000) with an estimated mass of 103 kDa. Since Ad3K is 69 kDa and EC2-3 is 27 kDa, we can exclude the presence of two or more receptors per trimeric fibre knob. The most likely explanation for a complex at 103 kDa is that only one receptor molecule is bound per trimeric knob (theoretical MW = 96 kDa). The 7% deviation is slightly above the 5% interval of confidence usually accepted with this technique. To support or reject this model an Analytical Ultra-Centrifugation (AUC) experiment was then performed. The Ad3K alone and Ad3K/EC2-3 complexes were run at two different concentrations. The shift between those two species was obvious with Ad3K/EC2-3 displaying a sedimentation coefficient of 5.40 S instead of 4.41 S for the fibre knob alone (Fig. 6B). More importantly, MW calculations from AUC data enabled the calculation of a molecular mass of 69.6 kDa for Ad3K and 95.1 kDa for the complex, thus confirming that only one receptor is bound per trimeric fibre knob. This observation represents a significant breakthrough in Adenovirus attachment to a protein cellular receptor. Indeed, up to now, the fibre of other adenovirus species described to interact with CAR, CD46 or sialic acid have been reported to exhibit three receptor binding sites per trimeric fibre^11,12,33^. The only notable exception to date lies on the interaction of HAd37 to the GD1a glycan^34^ for which a 1:1 (fibre:GD1a) interaction has also been reported. This disialylated hexasaccharide molecule carries two sialic acids which occupy two out of the three binding sites available in the trimer. Our finding for HAd3 binding to DSG2 clearly suggests that DSG2-interacting-Ads use a different strategy of attachment to the receptor and/or spreading as compared to other HAds. It is of note that free trimeric HAd2-fibre has been reported to disrupt CAR-mediated intercellular adhesion on its own^31^. In contrast, HAd3 fibre alone is not able to disrupt desmosomes and must be displayed on a scaffold of 12 penton bases (*i*.*e* virion or Pt-Dd) to trigger cell remodelling^9,22^. Even though our work has been performed with soluble DSG2 constructs, this may suggest that the 1:1 ratio (fibre:receptor) does not enable sufficient signal transduction in DSG2 integrated into the membrane and that dodecamerisation of the fibre in DSG2-interacting-HAds has evolved to overcome this limitation. Taken together, these results provide a molecular basis for Ad3K-DSG2 interactions and suggest that designing of HAd3-based therapeutics should take into account the novel stoichiometry of HAd3-receptor interactions. Increasing the numbers of Ad3Ks displayed on the Junction-Opener (JO) therapeutic is an attractive way to optimise junction-opening efficacy. Moreover, this biochemical and biophysical work provides a foundation for structural characterization of Ad3K in complex with DSG2 by either protein crystallographic or electron microscopy methods. As a longer-term goal, the identification of the residues from both partners involved in the interaction will allow the design of new HAd3-derived vectors with modified DSG2 tropism by incorporating rationally designed mutations in the HAd3 fibre knob.

## Methods

### Mammalian protein production

#### Construction of plasmids expressing His-DSG2

Coding sequence for human DSG2-cadherin domains EC1-4 (Ala50–Asp494 of the native protein, Swiss-Prot: Q14126.2) was amplified by PCR from the plasmid LV41-DSG2^35^ and cloned into the expression vector pHL-sec using the restriction sites AgeI/KpnI. pHL-sec was a gift from Edith Yvonne Jones (Addgene plasmid # 99845). It contains a Kozak sequence, a secretion signal sequence and a C-terminal hexa histidine tag^36^. Coding sequences for human DSG2-cadherin domains EC1-3, EC2-3 and EC3 (respectively Ala50–Ile386, Val149-Ile386, Ile262-Ile386 of the native protein) were amplified by PCR from the plasmid pHL-sec-DSG2 EC1-4 and inserted into pHL-sec using the restriction sites AgeI/KpnI.

#### Transient transfection

For protein expression, human embryonal kidney HEK293 F cells were maintained in FreeStyle™ 293 Expression medium (Gibco®, Thermo Fisher Scientific) in vented Erlenmeyer flasks (Corning) at 120 rpm, in a 37 °C, 5% CO2, humidified incubator. Cells were transiently transfected in flasks using Lipofectamine 2000 (LifeTechnologies) according to the manufacturer’s manual. 1 or 2 days before transfection, cells were seeded in fresh culture medium at 0.5 × 10^6^ cells/mL. When the density reached approximately 1 × 10^6^ cells/ml the cells were transfected. DNA was added to Lipofectamine 2000 in a 1:2.5 ratio, incubated at room temperature for 25 min and added to the cells. Supernatants were recovered 3 days post transfection by centrifugation at 1500 × g, 10 min at 4 °C. cOmplete™ EDTA-free Protease Inhibitor Cocktail (Roche Diagnostics) was added to the cleared supernatant prior to storage at −20 °C.

#### Purification of culture supernatants

Supernatants were pre-conditioned by the addition of sodium chloride, calcium chloride and imidazole to give final concentrations of 500 mM, 3 mM and 10 mM respectively. His-tagged proteins were extracted from the medium by affinity purification by loading the supernatants onto a 1 ml His GraviTrap prepacked columns (GE Healthcare), pre-equilibrated with 20 column volumes (CV) of 500 mM sodium chloride, 3 mM calcium chloride and 10 mM imidazole, 20 mM Tris-Cl pH8.0. The columns were then washed with 20 CV of the same buffer to remove non-specifically bound material. The second wash of the affinity columns was performed with the same buffer with increased imidazole concentration (12.5 mM). Proteins were eluted with 10 column volumes of the same buffer with 100 mM imidazole. Eluted proteins were concentrated and injected onto a Superdex™ 200 Increase 10/300 GL column (GE Healthcare) that had been equilibrated with gel filtration buffer (150 mM NaCl, 3 mM CaCl2, 10 mM Tris-Cl, pH8.0). After analysis of peak fractions by SDS-PAGE, proteins were concentrated at concentrations ranging from 2 to 6 mg/ml.

### Bacterial protein production

#### Construction of plasmids expressing His-Ad3K and His-Ad3K/MBP-DSG2

Coding sequence of the HAd3 fibre knob (Asn124 to Asp319 of the full-length protein, Genbank: ABB17809.1) was amplified from pQE-Ad3knob^13^. The PCR fragments were cloned into the pETDuet-1 plasmid (Novagen), downstream an N-terminal hexa histidine tag, for either single or co-expression use.

Coding sequences for human DSG2-cadherin domains EC2-3 and EC3 were amplified from the plasmid pHL-sec-DSG2 EC1-4 by PCR and introduced into the pETM-40 vector (EMBL), downstream of the maltose binding protein (MBP) gene. The sequences encoding the MBP/DSG2 EC2-3 or MBP/DSG2 EC3 fusion proteins were then amplified and cloned into the second multiple cloning site of the pETDuet-1 plasmid already encoding the HAd3 knob.

#### Expression and purification of His-Ad3K and His-Ad3K/MBP-DSG2

For protein expression, *E*. *coli* Rosetta™(DE3)pLysS cells (Novagen) were transformed with pETDuet-Ad3K, pETDuet-Ad3K/DSG2 EC2-3 or pETDuet-Ad3K/DSG2 EC3 vectors. A single colony was inoculated into LB medium containing carbenicillin (50 µg.mL−1) and chloramphenicol (34 µg.mL−1) and grown overnight at 37 °C. The culture was subsequently diluted to 1/100th into LB medium supplemented with carbenicillin and chloramphenicol. Bacteria were grown until OD600 reached 0.4–0.6. To induce protein expression, 1 mM of isopropyl β-D-1-thiogalactopyranoside was added to the culture which was transferred to 18 °C. After 18 h cells were harvested by centrifugation at 6,000 g for 15 min. Pelleted bacteria were resuspended in lysis buffer (500 mM NaCl, 3 mM CaCl_2_, 10 mM Imidazole, 20 mM Tris-Cl, pH8.0 and cOmplete™ EDTA-free Protease Inhibitor Cocktail) and lysed by sonication at 4 °C for 5 minutes 500 ms pulse. Cell debris was spun down after a centrifugation step at 39,200 g (JA20 rotor) for 30 min at 4 °C.

Clarified supernatants were then loaded onto a 1 ml His GraviTrap prepacked column (GE Healthcare) equilibrated with 20 CV of 500 mM sodium chloride, 3 mM calcium chloride and 10 mM imidazole, 20 mM, Tris-Cl pH8.0. The column was washed with 10 CV of equilibration buffer and proteins were eluted with the same buffer containing 200 mM imidazole.

Fractions containing the His-Ad3K/MBP-DSG2 EC2-3 or His-Ad3K/MBP-DSG2 EC3 complexes were pooled and treated 48 h at room temperature with Tobacco Etch Virus (TEV) protease at a ratio of 1:20 (w/w) during the dialysis step. To remove the cleaved Maltose Binding Protein (MBP), the His-Ad3K/DSG2 EC2-3 or His-Ad3K/DSG2 EC3 complexes were loaded again onto a 1 ml His GraviTrap prepacked column (GE Healthcare) and eluted as described above.

His-Ad3K alone, His-Ad3K/DSG2 EC2-3 and His-Ad3K/DSG2 EC3 complexes were further injected onto a Superdex™ 200 Increase 10/300 GL column (GE Healthcare) that had been equilibrated with gel filtration buffer (150 mM NaCl, 3 mM CaCl2, 10 mM Tris-Cl pH8.0). After analysis of peak fractions by SDS-PAGE, proteins were concentrated at concentrations ranging from 3 to 7 mg/ml.

### Surface Plasmon Resonance binding experiments (SPR)

Surface plasmon resonance binding experiments were performed at 25 °C using a BIAcore 3000 instrument (GE Healthcare) equipped with a CM4 sensor chip (GE Healthcare). DSG2 EC1-4, DSG2 EC1-2, DSG2 EC3-4 were diluted to 10 μg/ml in 10 mM sodium acetate, pH 4.5, and immobilized (3000–4000 RU) in 10 mM HEPES, 145 mM NaCl, 2 mM CaCl_2_, 0.005% surfactant P20, pH 7.4 (HBS-P calcium buffer) using the amine coupling chemistry according to the manufacturer instructions (GE Healthcare). Increasing concentrations of Pt-Dd were injected over the captured surfaces and binding was measured in HBS-P calcium buffer at a flow rate of 15 μl/min. Surfaces were regenerated by pulse injections of 5 mM EDTA.

### Analysis of protein-protein interactions by Size Exclusion Chromatography (SEC)

The interaction between Ad3K and DSG2 was investigated by size exclusion chromatography. The proteins were mixed in a 3:1 ratio (3 monomeric DSG2 to 1 trimeric Ad3K) in gel filtration buffer (150 mM NaCl, 3 mM CaCl2, 10 mM Tris-Cl pH8.0) and incubate overnight at 4 °C. The interaction of the proteins was analysed on a Superdex™ 200 Increase 10/300 GL column (GE Healthcare). The samples were centrifuged (3 min, 4 °C, 12,000 × g) and loaded onto the column using a 0.5 mL injection loop. The flow rate was 0.4 mL/min and the elution profile of the proteins was recorded at both 280 and 260 nm.

### Negative-stain electron microscopy

The standard mica-carbon preparation was used with sample at 50 ng/ml. Grids were stained by Uranyl acetate and observed at 100 kV on a Tecnai T12 microscope. 2D class averages were calculated by 2 cycles of classification from 13.143 and 13.149 original particles for Ad3K and Ad3K + EC2-3, respectively.

### Size Exclusion Chromatography coupled to Multi-Angle Laser Light Scattering experiments (SEC-MALLS)

SEC-MALLS experiments were performed using a A DAWN-Heleos II detector (Wyatt Technology) with a 690-nm wavelength laser in combination with an Optilab T-rEX Refractive index detector (Wyatt Technology). 50 μL of His-Ad3K/DSG2 EC2-3 at 1 mg/mL was injected over a Superdex™ 200 Increase 10/300 GL column (GE Healthcare) pre-equilibrated with gel filtration buffer (150 mM NaCl, 3 mM CaCl2, 10 mM Tris-Cl pH8.0) at 0.5 mL/min. A value of 0.185 mL/g was assumed for the dn/dc ratio of the protein. Astra software (Wyatt Technology) was used to analyze the data and to determine the weight-averaged molar masses (Mw).

### Sedimentation Equilibrium by Analytical Ultracentrifugation Experiments (SE-AUC)

Sedimentation velocity (SV) experiments were performed on a Beckman Coulter XL-I analytical ultracentrifuge with a An50Ti rotor. 3 mm or 12 mm pathlength 2-sector centerpieces cells, equipped with sapphire windows, were filled with 150 ul or 450 ul of sample respectively and centrifuged at 42,000 rpm at 20 °C. Sedimentation profiles were acquired using absorbance at 280 nm and interference optics.

Analysis and fitting of the data was performed using the software SedFit and Gussi. All s values obtained with the c(s) distribution in 3 mM CaCl2, 10 mM Tris pH 8.0, 150 mM NaCl were converted to s20,W with SEDNTERP (version 20130813 Beta) using the measured density (1.005 g/ml) and viscosity (1.019 cp) of this buffer.

## Electronic supplementary material

Supplementary Information
