# Supplementary material for: Mapping of Adenovirus of serotype 3 fibre interaction to desmoglein 2 revealed a novel ‘non-classical’ mechanism of viral receptor engagement
Source: Sci Rep. 2018 May 30;8:8381. doi: 10.1038/s41598-018-26871-x (PMC5976663; doi:10.1038/s41598-018-26871-x)
Supplement: Supplementary file 1 — Supplementary Information [file 41598_2018_26871_MOESM1_ESM.pdf]

Supplementary data

Mapping of Adenovirus of serotype 3 fibre interaction to desmoglein 2 revealed a novel 'non-classical' mechanism of viral receptor engagement.

Emilie Vassal-Stermann[1] ; Manon Mottet[1] ; Corinne Ducournau[1,2] ; Frédéric Iseni[2] ; Charles Vragniau[1] ; Hongjie Wang[3], Chloe Zubieta[4] ; André Lieber[3*] and Pascal Fender[1*]

Supplementary Figure S1

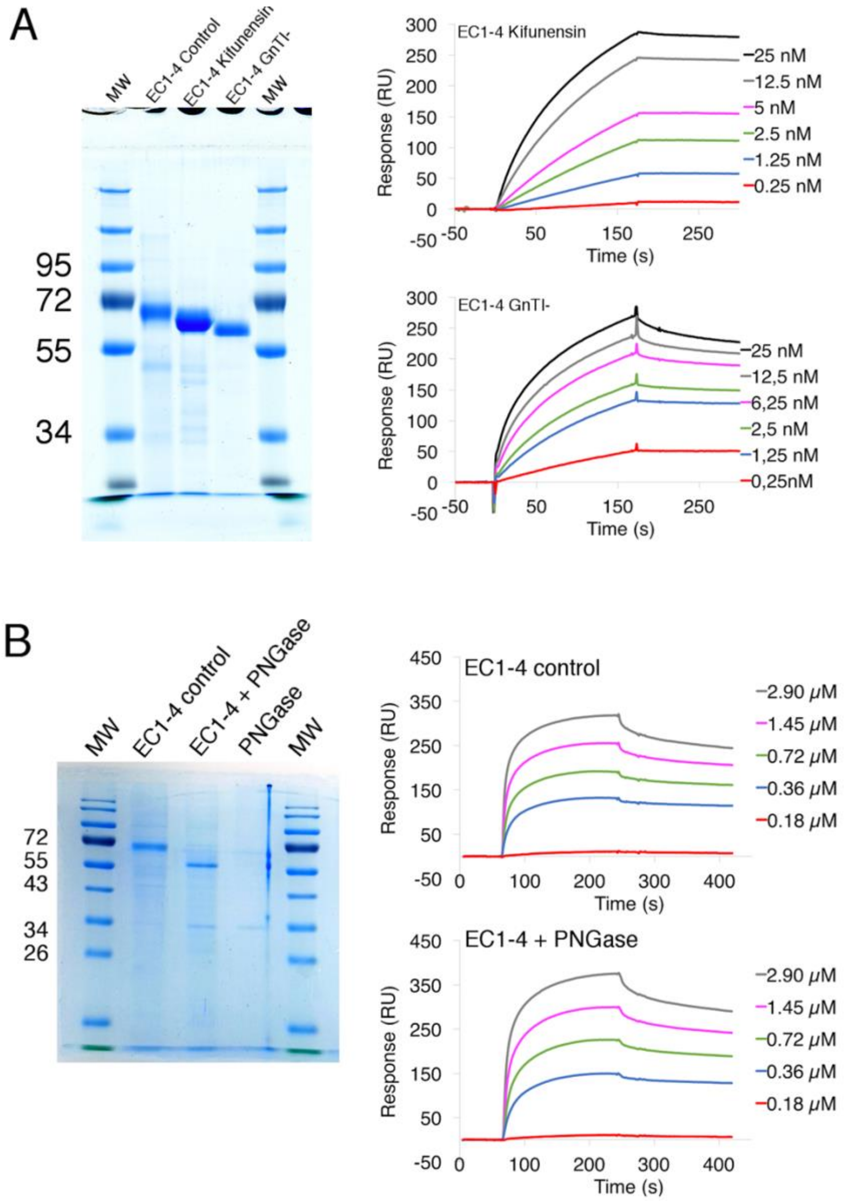

Legend to Supplementary Figure S1: De-glycosylation studies. (A) Full-length gel corresponding to the cropped image presented in Fig. 1B. EC1-4 protein expressed in 293F cell line with or without 10 μg.mL^{-1} of Kifunensin and EC1-4 protein expressed in 293 'GnTI^{-}' cell line (Glycosyl N-Transferase negative: ATCC CRL-3022) were run on SDS-PAGE. A shift in migration is observed when EC1-4 glycosylation is controlled. Surface plasmon resonance (SPR) analysis with those proteins immobilized into the sensorchip shows that Pt-Dd (HAd3 dodecahedron with 12 fibres) binding is not affected by the glycosylation modifications (B) Further de-glycosylation of EC1-4 expressed in 293F cell line was performed with the Peptide N-Glycosidase F (PNGaseF; Sigma-Aldrich) cleaving the whole glycan pattern from the aspargine residues. SDS-PAGE shows the shift in migration of the de-glycosylated EC1-4. SPR experiment performed with immobilized EC1-4 either glycosylated (upper sensorgrams) or deglycosylated (lower sensorgrams) demonstrates that Ad3K binding is not affected by the glycosylation removal from EC1-4.

Supplementary Figure S2

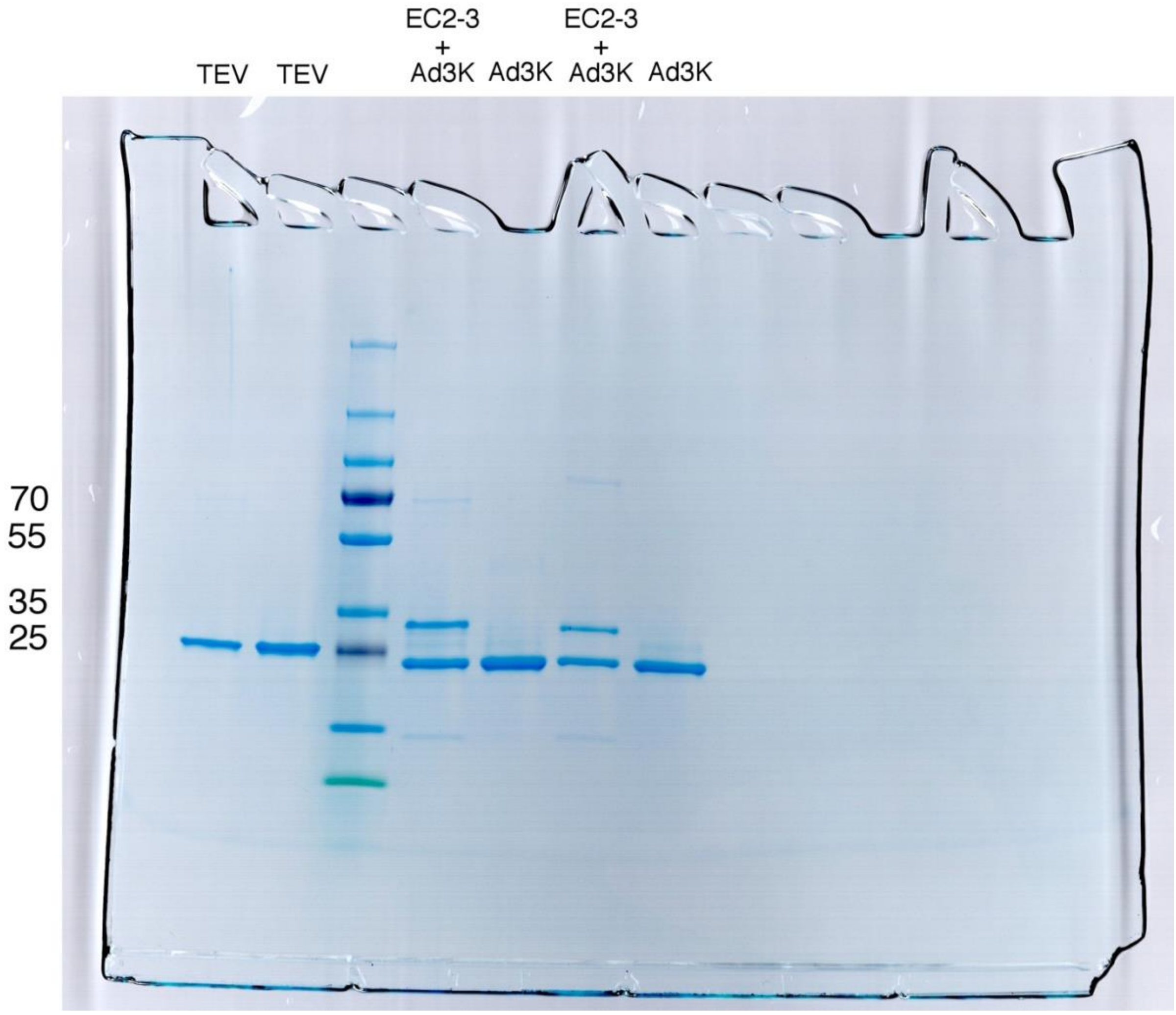

Legend to Supplemenatry Figure S2 : Full-length gel corresponding to the cropped image presented in Fig. 5A inset. Lanes 1 and 2 : TEV protease 1 and 2μg, respectively ; Lane 3 : MW Standard ; Lane 4 and 6 : EC2-3 + Ad3K complex after MBP removal (2μg and 1μg, respectively) ; Lanes 5 and 7 : Ad3K alone (2 and 1μg respectively).
